# Vascular ultrasound diagnosis and clinical implications of aberrant anterior tibial artery: case report

**DOI:** 10.1590/1677-5449.202400302

**Published:** 2024-12-09

**Authors:** Mariana Jordão França, Luciana Akemi Takahashi, Graciliano José França

**Affiliations:** 1 Universidade Positivo – UP, Curitiba, PR, Brasil.; 2 Universidade Federal do Paraná – UFPR, Complexo do Hospital de Clínicas – CHC, Curitiba, PR, Brasil.

**Keywords:** Doppler ultrasound, anterior tibial artery, anatomical variation, case report

## Abstract

The lower limb is vascularized by the femoral artery, which continues as the popliteal artery. After the distal margin of the popliteus muscle, the popliteal artery divides into the anterior and posterior tibial arteries. Anatomical variations in the bifurcation of the popliteal artery are frequent. One such variation is the aberrant anterior tibial artery, in which the artery runs anterior to the popliteus muscle instead of following the normal dorsal course. We report a case in which this anatomical variation was diagnosed with vascular ultrasound in a patient complaining of intermittent claudication in the left lower limb. The examination ruled out any possibility of obstructive arterial injury and identified a high bifurcation of the popliteal artery and the anterior tibial artery running anterior to the popliteus muscle and the tibial cortex. This anomalous variation is of great clinical importance, especially in surgical procedures involving the knee. Surgical injury to these arteries can lead to formation of pseudoaneurysm, compartment syndrome, or necrosis and can even result in limb amputation.

## INTRODUCTION

The popliteal artery is the continuation of the femoral artery and is involved in vascularization of the lower limb. Its downward course passes lateral to the opening of the adductor magnus muscle, passing through the adductor canal and then running obliquely to the distal margin of the popliteus muscle. At the margin of the popliteus muscle, the popliteal artery divides into the anterior and posterior tibial arteries.^1^ Beyond this bifurcation, the anterior tibial artery tends to follow a path dorsal to the popliteus muscle, passing between the heads of the tibialis posterior muscle and through an oval opening in the proximal interosseous membrane. Reaching the extensor portion of the leg, the artery travels medial to the head of the fibula and descends deep to the fibular nerve. After passing the ankle, it continues along the dorsum of the foot, as the dorsalis pedis artery.^1,2^ Variations in the terminal branches of the popliteal artery are related to vascular embryology and are linked to combinations of the sciatic/axillary artery and the femoral artery.^1,3^

**Figure 1:**
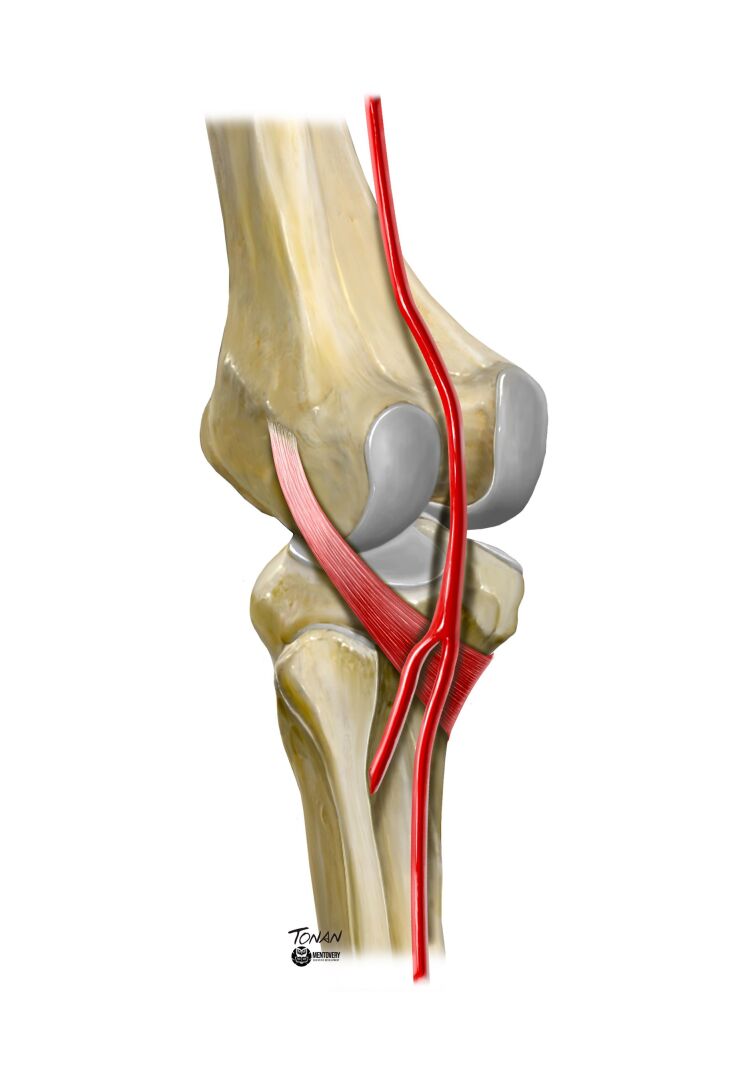
Illustration of the normal anatomy of the lower limb. The anterior tibial artery follows its usual course, dorsal to the popliteus muscle. Illustration by Rodrigo Tonan, Mentovery.

**Figure 2:**
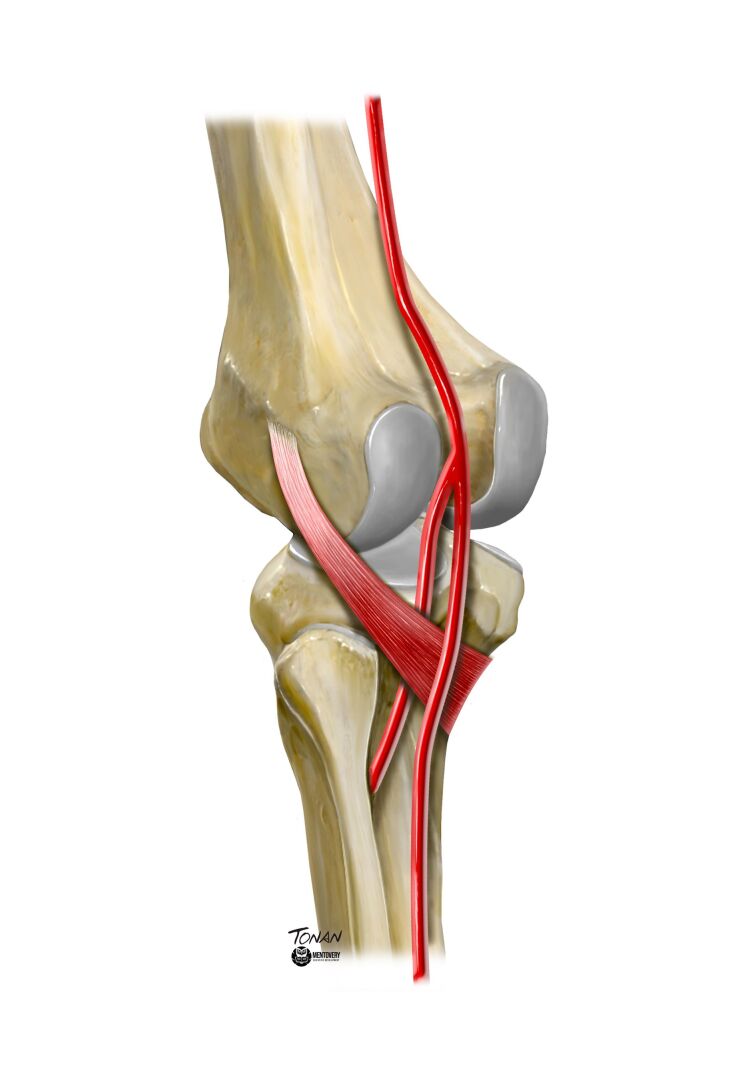
Illustration of variant anatomy of the lower limb. Aberrant anterior tibial artery following a variant course, anterior to the ventral margin of the popliteus muscle, and to the posterior tibial cortex. Illustration by Rodrigo Tonan, Mentovery.

Variations are relatively common and are present in 10% of the population.^4,5^ There are several different variations, including high origin of the anterior tibial artery. There is a subvariant of this case, known as aberrant anterior tibial artery, in which the artery passes anterior to the popliteus muscle, along its ventral margin, and to the posterior tibial cortex (Figures 1 and 2).^4^ This variant has been reported in autopsies and magnetic resonance, computed tomography angiography, and ultrasound scans.^4^ We report the case of a patient who underwent arterial Doppler ultrasound of the lower limbs after complaining of intermittent claudication, in whom the aberrant anterior tibial artery anatomic variant was diagnosed on the basis of the vascular ultrasound findings.

The study was approved by the Ethics Committee at our institution (opinion number 5.806.094). A free and informed consent form for studies involving human beings was signed.

## CASE DESCRIPTION

**Figure 3:**
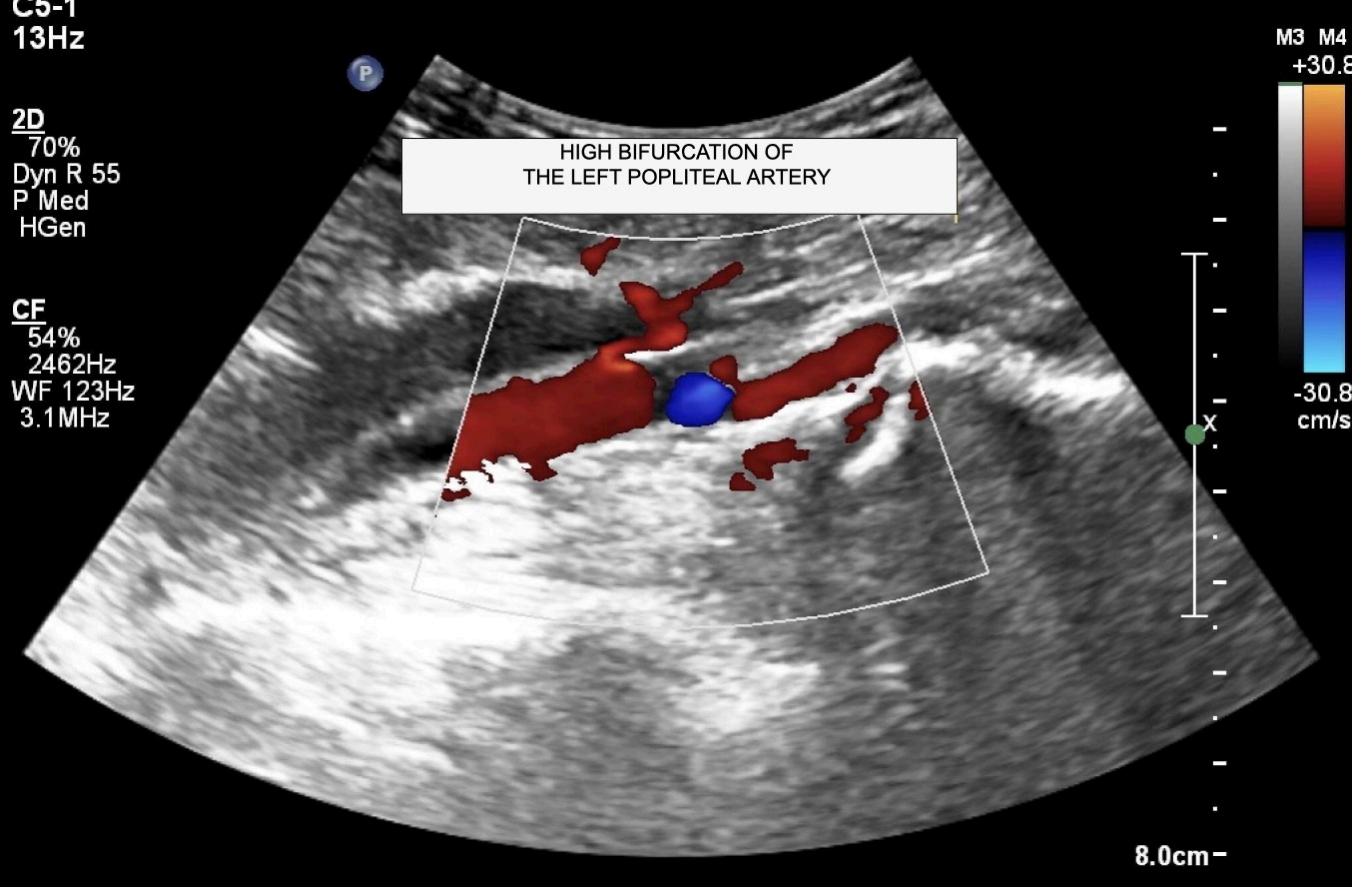
Doppler ultrasonography of the left lower limb in the longitudinal plane, showing high bifurcation of the popliteal artery.

**Figure 4:**
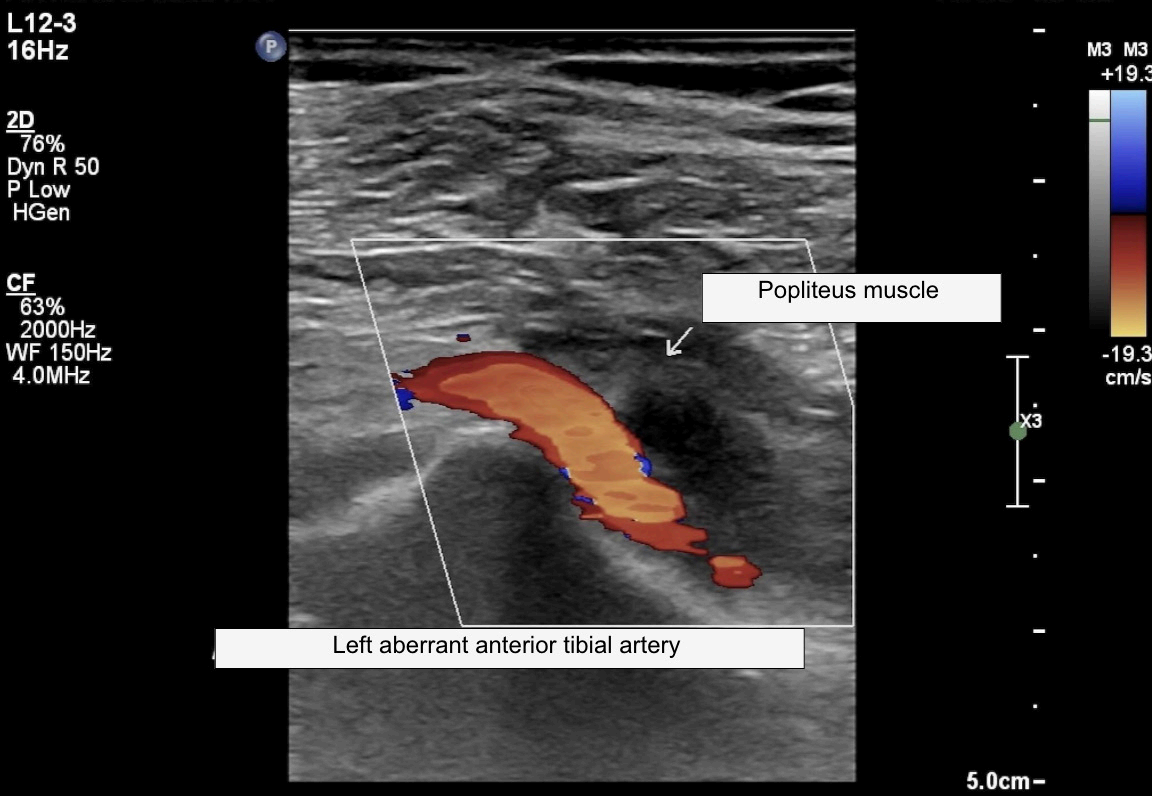
Doppler ultrasonography of the left lower limb in the longitudinal plane, showing the aberrant anterior tibial artery anatomic variant. The artery passes anterior to the popliteus muscle.

The patient was a 66-year-old male with no comorbidities who was referred to the vascular clinic after complaining of intermittent claudication in the left lower limb. He reported stabbing pains in the left leg when walking a few blocks, which subsided with rest. Physical examination found no changes to color, temperature, elasticity, or sensitivity of the limb. The femoral, popliteal, posterior tibial, and pedal pulses were all present in both limbs. Vascular ultrasound was ordered to support diagnosis. The Doppler scan was performed with the patient in the supine position, with the head raised, using a high frequency linear transducer to assess the arterial anatomy of the left lower limb. Images were acquired in transverse and longitudinal planes and spectral analysis was conducted. The ultrasound examination ruled out any possibility whatsoever of obstructive arterial injuries. A high bifurcation of the popliteal artery was observed in the left lower limb (Figure 3) and an aberrant anterior tibial artery was identified (Figure 4). There was no anatomic evidence of a similar variant in the contralateral lower limb. The patient was discharged after the vascular assessment and instructed to disclose the existence of the anatomic variant before any medical procedure involving the left lower limb, especially knee surgery.

## DISCUSSION

In the embryo, arterial supply to the lower limb is provided by the axial and femoral arteries. As the embryo develops, these arteries anastomose. The axial artery continues as the deep popliteal artery, which courses ventral to the popliteus muscle. This artery also gives rise to the primitive anterior tibial artery and the peroneal arteries, which pass dorsal to the popliteus muscle. Fusion of these primordial vessels forms part of the popliteal artery, with the superficial branch dorsal to the muscle and the deep branch ventral to the muscle. Communication between the superficial and deep popliteal arteries gives rise to the anterior tibial artery. During the third month of gestation, the deep branch of the popliteal artery involutes, resulting in the adult arterial anatomy.^3^

According to Senior et al.,^6^ failures to achieve correct communication between the superficial and deep branches of the popliteal artery cause high division of the popliteal artery, which is responsible for formation of the aberrant anterior tibial artery as a result of persistence of the deep popliteal artery and failure of development of the medial communicating branch.^4,7^ The prevalence of this anatomic variant ranges from 1.8 to 6%.^3,8^

Kim et al.^9^ has proposed a unified classification of variations of the popliteal artery: type I is the normal pattern of division, type II is high division of the popliteal artery, and type III is presence of a hypoplastic branch. Type II also has a subclassification: IIA1, in which the vessel courses posterior to the popliteus muscle, and IIA2, in which the artery courses between the anterior aspect of the popliteus muscle and the posterior tibial cortex. The aberrant anterior tibial artery is classified as type IIA2.

Injuries to the aberrant anterior tibial artery have been reported in relation to arthroscopy, total osteotomy, lateral meniscus repair, reconstruction of the posterior cruciate ligament, and knee replacement surgeries. Complications of arterial injury include pseudoaneurysm formation, compartment syndrome, necrosis, and even limb amputation.^4,10^ Some studies have found that placing the knee in flexion at 90 degrees during surgery protects the vessel. This is because the artery tends to move away from the posterior part of the tibia during knee flexion, thereby reducing the risk of injury.^3^ However, this technique does not work in anatomic variant cases in which the aberrant anterior tibial artery is present, because the vessel is pressed against the tibia by the popliteus muscle. In these anatomic variant cases, it is recommended that safe surgical techniques should be used. In order to avoid injuries, Ahn et al.^11^ recommend arthroscopy limited to the posterior capsule of the posterior cruciate ligament, increasing the distance between the popliteal artery and the posterior tibial cortex. When performing lateral meniscus repair, the “inside out” method is preferable because of the position of the posterior capsule and direct visualization of the aberrant vessel. For total knee replacement, the tibia should only be cut after subperiostial dissection of the posterior tibial cortex and adequate protection of the neurovascular structures.^3^

Doppler ultrasound is a precise evaluation method for investigation of aberrant anterior tibial artery cases, despite being limited to partial assessment of the vascular anatomy of the knee.^8^ Moreover, since it is a rapid, inexpensive, and noninvasive examination that is widely available, it can be used for preoperative investigation of patients undergoing knee surgery, helping to rule out the possibility of anatomic variants, such as aberrant anterior tibial artery, and avert possible operative complications.

## CONCLUSIONS

The case presented, of a patient complaining of intermittent claudication in the left lower limb, clearly demonstrates the importance of imaging exams to rule out the possibility of anatomic variants. The method used in this context, vascular ultrasound, is a precise evaluation tool for investigation of aberrant anterior tibial artery cases, despite being limited to partial assessment of the vascular anatomy of the knee.^8^ Moreover, since it is a rapid, inexpensive, and noninvasive examination that is widely available, it can be used for preoperative investigation of patients undergoing knee surgery. Ruling out the possibility of anatomic variants, such as aberrant anterior tibial artery, before surgical procedures yields a better understanding of the patient’s operative anatomy and helps to avoid possible complications, such as pseudoaneurysm formation, compartment syndrome, necrosis, or even limb amputation, especially in arthroscopy, osteotomy total, lateral meniscus repair, reconstruction of the posterior cruciate ligament, and knee replacement surgeries.

## CONSENT FORM

The patient signed free and informed consent to publications of ultrasonographic images and the case description.

## REFERENCES
