# Supplementary material for: Vascular ultrasound diagnosis and clinical implications of aberrant anterior tibial artery: case report
Source: J Vasc Bras. 2024 Dec 9;23:e20240030. doi: 10.1590/1677-5449.202400302 (PMC11702971; doi:10.1590/1677-5449.202400302)
Supplement: Translation

# Translation: Diagnóstico e implicações clínicas de variação anômala da artéria tibial anterior por ultrassom vascular: relato de caso

**DOI:** 10.1590/1677-5449.202400301

**Authors:** Mariana Jordão França, Luciana Akemi Takahashi, Graciliano José França

**Affiliations:** 1 Universidade Positivo – UP, Curitiba, PR, Brasil.; 2 Universidade Federal do Paraná – UFPR, Complexo do Hospital de Clínicas – CHC, Curitiba, PR, Brasil.

**Keywords:** ultrassom Doppler, artéria tibial anterior, variação anatômica, relato de caso

## Abstract

O membro inferior é vascularizado pela artéria femoral, a qual se continua em artéria poplítea. Após a borda distal do músculo poplíteo, a artéria poplítea se divide em artéria tibial anterior e posterior. Variações anatômicas na bifurcação da artéria poplítea ocorrem com frequência, e uma dessas variações é a artéria tibial anterior aberrante, na qual a artéria apresenta trajeto anterior ao músculo poplíteo em vez de ter percurso dorsal. Reportamos um diagnóstico dessa variação anatômica em um paciente com queixa de claudicação intermitente no membro inferior esquerdo. O exame de ultrassom vascular excluiu a possibilidade de qualquer lesão arterial obstrutiva e constatou uma bifurcação alta da artéria poplítea, além de identificar o percurso da artéria tibial anterior aberrante, posicionada anteriormente ao músculo poplíteo e ao córtex tibial posterior. Essa variação anômala apresenta grande importância clínica, especialmente em procedimentos cirúrgicos no joelho. Lesões cirúrgicas dessa artéria podem levar à formação de pseudoaneurisma, síndrome compartimental, necrose ou até mesmo amputação do membro.

## INTRODUÇÃO

A vascularização do membro inferior envolve a artéria poplítea, que é a continuação da artéria femoral. Seu percurso descendente passa lateralmente à abertura do músculo adutor magno, atravessando o canal dos adutores e se inclinando de forma oblíqua até a borda distal do músculo poplíteo. Na margem do músculo poplíteo, a artéria poplítea se divide em artérias tibial anterior e posterior^1^ . Após a bifurcação, a artéria tibial anterior tende a percorrer um percurso dorsal ao músculo poplíteo, passando entre as cabeças do músculo tibial posterior e pela abertura oval da parte proximal da membrana interóssea. Ao chegar na parte extensora da perna, a artéria passa medial à cabeça da fíbula e desce profundamente ao nervo fibular. Após passar o tornozelo, continua no dorso do pé como artéria pediosa dorsal^1,2^ . Variações nos ramos terminais da artéria poplítea têm relação com a embriologia vascular, tendo ligação com as combinações da artéria isquiática/axilar e artéria femoral^1,3^ .

As variações são relativamente comuns, estando presentes em 10% da população^4,5^ . Existem diferentes variações, entre elas a origem alta da artéria tibial anterior. Nesse caso, existe uma subvariante conhecida como artéria tibial anterior aberrante, na qual o trajeto arterial passa anteriormente ao músculo poplíteo, ao longo de sua margem ventral, e ao córtex tibial posterior (Figuras 1 e 2)^4^ . Essa variação foi reportada em autópsias, ressonâncias magnéticas, angiotomografias e ultrassonografias^4^ . Reportamos o caso de um paciente em que foi solicitado um Doppler arterial de membros inferiores após queixa de claudicação intermitente e, a partir do ultrassom vascular, diagnosticada a presença da variação anatômica da artéria tibial anterior aberrante.

**Figura 1:** Ilustração da anatomia usual do membro inferior. Artéria tibial anterior com trajeto usual e percurso dorsal ao músculo poplíteo. Ilustração de autoria de Mentovery Rodrigo Tonan.

**Figura 2:** Ilustração da anatomia variante do membro inferior. Artéria tibial anterior aberrante com trajeto variante arterial anterior ao músculo poplíteo, em sua margem ventral, e ao córtex tibial posterior. Ilustração de autoria de Mentovery Rodrigo Tonan.

O estudo foi aprovado pelo Comitê de Ética de nossa instituição (parecer número 5.806.094). O termo de consentimento livre e esclarecido foi assinado para os estudos que envolvem seres humanos.

## DESCRIÇÃO DO CASO

O paciente, um homem de 66 anos, sem comorbidades, foi encaminhado para ambulatório da vascular após queixa de claudicação intermitente em membro inferior esquerdo. Relatava dores e fisgadas na perna esquerda ao caminhar algumas quadras, as quais eram aliviadas pelo repouso subsequente. No exame físico, não havia nenhum sinal de alteração de cor, temperatura, elasticidade e sensibilidade no membro. Os pulsos femoral, poplíteo, tibial posterior e pedioso estavam presentes em ambos os membros. Para complementação diagnóstica, foi solicitado um ultrassom vascular. O Doppler foi realizado com o paciente em decúbito dorsal, com a cabeceira elevada, utilizando-se um transdutor linear de alta frequência, visando avaliar a anatomia arterial do membro inferior esquerdo. Foram realizados cortes transversais, longitudinais e análise espectral. O ultrassom excluiu a possibilidade de qualquer lesão arterial obstrutiva. Foi constatada uma bifurcação alta da artéria poplítea (Figura 3) e a presença da artéria tibial anterior aberrante no membro inferior esquerdo (Figura 4). Não havia evidência anatômica de variação similar no membro inferior contralateral. O paciente foi liberado após avaliação da vascular e orientado a informar a presença dessa variação anatômica em qualquer procedimento médico no membro inferior esquerdo, principalmente em caso de cirurgia no joelho.

**Figura 3:** Eco-Doppler do membro inferior esquerdo em corte longitudinal, demonstrando a bifurcação alta da artéria poplítea.

**Figura 4:** Eco-Doppler do membro inferior esquerdo em corte longitudinal mostrando a variação anatômica da artéria tibial anterior aberrante. A artéria passa anteriormente ao músculo poplíteo.

## DISCUSSÃO

O suprimento arterial embriológico do membro inferior é realizado pelas artérias axial e femoral. Ao decorrer do desenvolvimento, essas artérias se anastomosam. A artéria axial se continua como artéria poplítea profunda, a qual tem percurso ventral ao músculo poplíteo. Essa artéria também origina a artéria tibial anterior primitiva e as artérias peroneais, que passam dorsalmente ao músculo poplíteo. A fusão desses vasos primordiais forma parte da artéria poplítea, sendo o ramo superficial dorsal ao músculo, enquanto o ramo profundo se localiza ventralmente ao músculo. A comunicação dos ramos poplíteo superficial e profundo leva à origem da artéria tibial anterior. No terceiro mês de gestação, o ramo profundo da artéria poplítea é involuído, resultando em uma anatomia arterial adulta^3^ .

De acordo com Senior et al.^6^ , falhas na comunicação correta dos ramos superficial e profundo da artéria poplítea levam à alta divisão da artéria poplítea, a qual é responsável pela formação da artéria tibial anterior aberrante, que é a persistência da artéria poplítea profunda e a falha no desenvolvimento do ramo comunicante médio^4,7^ . A prevalência dessa variação anatômica varia entre 1,8 e 6%^3,8^ .

Kim et al.^9^ propôs uma classificação unificada das variações da artéria poplítea: o tipo I se refere à divisão normal, o tipo II à alta divisão da artéria poplítea e o tipo III à presença de ramo hipoplásico. Para o tipo II, ainda existe uma subclassificação: IIA1, onde o vaso tem percurso posterior ao músculo poplíteo, e IIA2, onde a artéria percorre entre a face anterior do músculo poplíteo e o córtex tibial posterior. A artéria tibial anterior aberrante é classificada como tipo IIA2.

Lesões na artéria tibial anterior aberrante foram reportadas em cirurgias de artroscopia, osteotomia total, reparo lateral de menisco, reconstrução do ligamento cruzado posterior e prótese de joelho. Algumas complicações de lesão arterial são formação de pseudoaneurisma, síndrome compartimental, necrose ou até mesmo amputação do membro^4,10^ . Alguns estudos constatam que a posição cirúrgica do joelho flexionado a 90 graus protege o vaso de lesões nos procedimentos cirúrgicos. Isso porque, com a flexão do joelho, a tendência da artéria é se afastar da parte posterior da tíbia, reduzindo assim o risco de lesão^3^ . Todavia, essa técnica não funciona em casos de variação anatômica com a presença da artéria tibial anterior aberrante, devido ao vaso estar pressionado contra a tíbia pelo músculo poplíteo. Nesses casos de variação anatômica, recomenda-se o uso de técnicas cirúrgicas seguras. Para evitar lesões, Ahn et al.^11^ recomendam artroscopia limitada à cápsula posterior do ligamento cruzado posterior, aumentando a distância entre a artéria poplítea e o córtex tibial posterior. Em caso de reparo de menisco lateral, o método “*inside out*” é preferível devido à posição da cápsula posterior e à visualização direta do vaso aberrante. Para próteses de joelho total, deve-se proceder com o corte da tíbia somente após a dissecação subperióstea do córtex tibial posterior e a proteção adequada das estruturas neurovasculares^3^ .

O ultrassom Doppler é um método de avaliação preciso em casos de investigação da artéria tibial anterior aberrante, apesar de sua limitação de avaliação parcial da anatomia vascular do joelho^8^ . Além disso, por ser um exame não invasivo, rápido, de baixo custo e fácil disponibilidade, pode servir como investigação pré-operatória em pacientes submetidos a cirurgias de joelho, ajudando a descartar qualquer possível variação anatômica, como a artéria tibial anterior aberrante, e a evitar possíveis complicações operatórias.

## CONCLUSÃO

O caso apresentado, de um paciente com queixa de claudicação intermitente em membro inferior esquerdo, deixa claro a importância de um exame de imagem para descartar qualquer possível variação anatômica. O método utilizado nesse cenário, o ultrassom vascular, é uma ferramenta de avaliação precisa em casos de investigação da artéria tibial anterior aberrante, apesar de sua limitação de avaliação parcial da anatomia vascular do joelho^8^ . Além disso, por ser um exame não invasivo, rápido, de baixo custo e fácil disponibilidade, pode servir como investigação pré-operatória em pacientes submetidos a cirurgias de joelho. Descartar qualquer possível variação anatômica, como a artéria tibial anterior aberrante, antes de procedimentos cirúrgicos, permite um melhor entendimento da anatomia operatória do paciente e ajuda a evitar possíveis complicações, como a formação de pseudoaneurisma, síndrome compartimental, necrose ou até mesmo amputação do membro, especialmente em cirurgias de artroscopia, osteotomia total, reparo lateral de menisco, reconstrução do ligamento cruzado posterior e prótese de joelho.

## TERMO DE CONSENTIMENTO

O paciente assinou o termo de consentimento livre e esclarecido para a divulgação de imagens ultrassonográficas e publicação do caso.
